# Effective antiprotease-antibiotic treatment of experimental anthrax

**DOI:** 10.1186/1471-2334-5-25

**Published:** 2005-04-08

**Authors:** Serguei G Popov, Taissia G Popova, Svetlana Hopkins, Raymond S Weinstein, Rebecca MacAfee, Karl J Fryxell, Vikas Chandhoke, Charles Bailey, Ken Alibek

**Affiliations:** 1 Advanced Biosystems, Inc., Manassas, VA, USA; 2 National Center for Biodefense, George Mason University, Manassas, VA, USA; 3 Potomac Hospital, Woodbridge, VA, USA; 4 Center for Biomedical Genomics & Informatics, Department of Molecular & Microbiology, George Mason University, Manassas, VA, USA; 5 Current affiliation: National Center for Biodefense, George Mason University, Manassas, VA, USA

## Abstract

**Background:**

Inhalation anthrax is characterized by a systemic spread of the challenge agent, *Bacillus anthracis*. It causes severe damage, including multiple hemorrhagic lesions, to host tissues and organs. It is widely believed that anthrax lethal toxin secreted by proliferating bacteria is a major cause of death, however, the pathology of intoxication in experimental animals is drastically different from that found during the infectious process. In order to close a gap between our understanding of anthrax molecular pathology and the most prominent clinical features of the infectious process we undertook bioinformatic and experimental analyses of potential proteolytic virulence factors of *B. anthracis *distinct from lethal toxin.

**Methods:**

Secreted proteins (other than lethal and edema toxins) produced by *B. anthracis *were tested for tissue-damaging activity and toxicity in mice. Chemical protease inhibitors and rabbit immune sera raised against *B. anthracis *proteases were used to treat mice challenged with *B. anthracis *(Sterne) spores.

**Results:**

*B. anthracis *strain delta Ames (pXO1^-^, pXO2^-^) producing no lethal and edema toxins secrets a number of metalloprotease virulence factors upon cultivation under aerobic conditions, including those with hemorrhagic, caseinolytic and collagenolytic activities, belonging to M4 and M9 thermolysin and bacterial collagenase families, respectively.

These factors are directly toxic to DBA/2 mice upon intratracheal administration at 0.5 mg/kg and higher doses. Chemical protease inhibitors (phosphoramidon and 1, 10-phenanthroline), as well as immune sera against M4 and M9 proteases of *B. anthracis*, were used to treat mice challenged with *B. anthracis *(Sterne) spores. These substances demonstrate a substantial protective efficacy in combination with ciprofloxacin therapy initiated as late as 48 h post spore challenge, compared to the antibiotic alone.

**Conclusion:**

Secreted proteolytic enzymes are important pathogenic factors of *B. anthrasis*, which can be considered as effective therapeutic targets in the development of anthrax treatment and prophylactic approaches complementing anti-lethal toxin therapy.

## Background

Inhalation anthrax is a severe, often fatal disease characterized by systemic spread of the challenge agent, *Bacillus anthracis*, which is capable of causing severe damage to host tissues and organs. Multiple hemorrhagic lesions in the mediastinum, mediastinal lymph nodes, bronchi, lungs, heart, spleen, liver, intestines, kidneys, adrenal glands, and/or central nervous system are typically found upon postmortem examination of patients who succumbed to inhalation anthrax. The most dramatic and potentially life-threatening changes were observed in the vascular system with a diffuse vasculitis extending from moderate sized arteries and veins down to the capillary level. The vasculitis was often associated with vessel destruction, especially of the smallest vessels, and was typically accompanied by massive necrosis in some tissues [1-3]. It is widely believed that anthrax lethal toxin (LT) secreted by proliferating bacteria is a major cause of death in man and in several other susceptible animal species [4]. However, the pathology of intoxication in experimental animals is drastically different from that found during the natural infectious process. Recent extensive analyses in mice and rats challenged with a highly purified lethal toxin [5,6] confirmed earlier observations [7] that toxin activity caused no gross pathology and almost solely manifested in hypoxic liver failure. In addition to lethal toxin, the hemorrhagic and other tissue-damaging factors elaborated by *B. anthracis *could play important virulence-enhancing roles but these factors have not yet been characterized. Early publications using culture filtrates of *B. anthracis *assumed that the observed effects of secreted substances were caused entirely by LT [8,9]. It is also possible that these other factors could themselves exhibit a direct lethal effect.

The capacity of bacteria to cause destruction of tissues, as well as other pathological consequences such as degradation of immunoglobulins, cytokines and complement, release of inflammatory mediators, or activation of host proteolytic enzymes, is attributed to a wide variety of secreted proteases [10], however in the case of *B. anthracis *the proteases of this microorganism, other than lethal factor, have attracted little attention in the scientific literature. The current study aimed to carry out an initial characterization of certain *B. anthracis *proteolytic enzymes and to obtain evidence on their possible pathogenic role in anthrax.

## Methods

### Microbial strains

The non-encapsulated *Bacillus anthracis *strain 34F2 (Sterne) [pXO1+, pXO2-] obtained from the Colorado Serum Company (Boulder, CO) was used in animal challenge experiments. The 50% lethal doses (LD_50_s) by the intraperitoneal (i.p.) route were established earlier (Popov *et al*., 2004) and the LD_50 _value for intraperitoneal challenge for DBA/2 mice was found to be 3 × 10^6 ^spores per mouse. The non-encapsulated, atoxigenic strain of *B. anthracis *(delta Ames) [pXO1-, pXO2-] was kindly provided by Dr. J. Shiloach (National Institutes of Health, Bethesda, MD). *B. cereus *strain ATCC #11778 and *B. subtilis *strain #23857 were purchased from American Type Culture Collection (Manassas, VA).

### Mice

Female DBA/2 mice (9 weeks old) were obtained from Taconic (Germantown, NY) and were used throughout the study.

### Reagents

The following substances were used in this study: ciprofloxacin (ICN Biomedicals, lot no.4913F), phosphoramidon disodium salt, and 1,10-phenanthroline (Sigma, MO), EDTA (GibcoBRL, CA), soybean trypsin inhibitor from Glycine max (Sigma, MO), thermolysin (EC 3.4.24.27) from *Bacillus thermoproteolyticus *(Sigma, MO). The fluorescently labeled casein and collagen type I for determination of proteolytic acivity were from Molecular Probes (OR). Zymogram gels were from Invitrogen (Carlsbad, CA). Lethal factor (LF) and protective antigen (PA) were from List Biological Laboratories (CA).

### Preparation of secreted proteins

Secreted substances were prepared by culturing *B. anthracis *(delta Ames) in LB media overnight. Cells were removed by centrifugation at 8000 g, and the supernatant was sterilized by filtration through 0.22 μm cellulose acetate filtration system (Corning, NY) and further concentrated 50-fold using Amicon Ultra15 centrifugal filter devices (10 K cut-off pore size) (Millipore, MA). The proteins were used immediately after preparation or were stored at 4°C for several days. Protein content was determined using Bradford reagent (Bio-Rad) with bovine serum albumin as standard. Slow reduction in the hemorrhagic activity was found upon storage within a week.

### Fractionation of culture supernatants

1 ml of *B. anthracis *culture supernatant (BACS) was loaded onto the size-exclusion Superdex column (25 × 60, Pharmacia Biotech) and was eluted with PBS (pH 7.4) with a flow rate of 2 ml/min. Fractions of eluate were concentrated to equal volumes using Centricon devices (Millipore, MA) with a 10 K cut-off pore size.

### Hemorrhages in femoral artery region

Mice were anesthetized by intraperitoneal injection of Avertin (2,2,2 tribromethanol, Aldrich) and 100 μl of secreted proteins (20 to 100 μg) were subcutaneously (s.c.) injected into the femoral artery region for observation of hemorrhagic changes after 3 to 15 h. In order to record hemorrhagic changes animals were anesthetized by i.p. injection of Avertin and the fur over the femoral artery region was removed to allow observation of a 1.5 to 2.5 cm^2 ^area of skin. It was photographed, and the size of the hemorrhagic spot was measured. In the experiments on the inhibition of hemorrhagic effect the secreted proteins were preincubated with specific antisera or protease inhibitors for 30 min on ice.

### Generation of antibodies against *B. anthracis *MPs

The Invitrogen (CA) custom service was used to obtain rabbit polyclonal sera against peptides conjugated with kallikrein (Table 1). Two animals were immunized by each conjugate. All six rabbit sera had ELISA titers ranging from 100,000 to 200,000. For generation of murine polyclonal antibodies against the M4 protease (BA3442) the C-terminal part of the gene encoding amino acids 248 to 532 was cloned into pTrcHis2 TOPO TA cloning vector (Invitrogen, CA). The recombinant protein containing a 6 × His tag was expressed in *E. coli *and purified using the Ni-NTA resin (Quiagen, CA). Mice were immunized with 50 μg of the protein emulsified in a complete Freund's adjuvant and were given two booster immunizations using an incomplete adjuvant with 2 week intervals. Serum was collected after two weeks since the last boost injection. In the skin hemorrhagic test described above, 30 μl of serum was able to completely suppress the hemorrhage caused by 30 μl of BACS.

**Table 1:** Sera against *B. anthracis *proteases

**Serum #**	**Protease family**	**Protein**	**Gene number**	**Antigen**	**Designation**
1	M4	Elastase-like neutral protease	BA3442	Recombinant polypeptide corresponding to the fragment 248–532.	M4EL
2	M9	Collagenase	BA0555, BA3299, BA3584	HEFTHYLQGRYEVPGL spanning the region of active center	M9Coll
3	M4	Neutral protease	BA5282, BA0599	DVIGHELTHAVTE spanning the region of active center	M4AC
4	M4	Neutral protease	BA2730	ADYTRGQGIETY distant from the active center	M4EP

### Intratracheal delivery of *B. anthracis *secreted proteins

Mice were anesthetized by i.p. injection of Avertin and a 24G angiogenic catheter (BD Biosciences, CA) was inserted into the trachea. 50 μl of experimental mixture, containing 10 to 100 μg of culture supernatant proteins were slowly injected through the catheter connected to a microsyringe. The angiogenic catheter was removed and animals were left for further observation. The untreated control group received the same volume of phosphate-buffered saline (PBS). A control group of 3 animals was injected with 50 μl of PBS solution of lethal toxin (100 μg PA+100 μg LF). In all experiments the rate of breathing was recorded every 10 min during the first 3 h following injection, and animals were observed for survival for 7 days.

### Detection of proteolytic activity of culture supernatants

Proteolitic activities of supernatants were measured using EnzChek Protease assay kit (Molecular Probes) and EnzChek Gelatinase/Collagenase assay kit (Molecular Probes) according to the manufacturer's protocols. Briefly, 5 μl of different dilutions of supernatant in 45 μl of digestion buffer were mixed with 50 μl of fluorescein-labeled substrate (casein or gelatin) and fluorescence intensity was measured at different time points using 485 nm excitation and 530 nm emission wavelengths.

### Treatment of spore-challenged mice

Mice used in all experiments were maintained under proper conditions with a 12-h light/dark cycle in accordance with IACUC standards in the animal facility of the Biocon, Inc. (Rockville, MD). Mice received food and water *ad libitum*. Groups of 10 mice were randomly assigned for challenge and were observed for survival and signs of disease. The animals were inoculated i.p. by 1 × 10^7 ^spores per mouse of Sterne strain. Treatment (i.p.) with phenanthroline (30 mg/kg), phosphoramidon (10 mg/kg), or rabbit sera (5 or 25 mg/kg) was carried out individually for each substance or in combination with ciprofloxacin at (50 mg/kg) once a day started at different time points post spore challenge and continued for 10 days. In all experiments the animals were monitored for survival for at least 12 days after termination of treatment.

### Statistical analysis

Kaplan-Meier open-end survival analysis was performed to compare results between treatment groups. Statistical significance was established as *P *< 0.05 using log-rank test.

## Results

### Genomic analysis of *B. anthracis *secreted proteins as potential virulence factors

In order to evaluate a pathogenic potential attributed to the *B. anthracis *proteins other than known lethal and edema toxins we used a nontoxigenic and nonencapsulated strain of *B. anthracis *(delta Ames), which is a parental Ames strain cured of both plasmids, pXO1 and pXO2. The substances secreted by vegetative *B. anthracis *cells seem to be the most promising candidates, as is the case for many bacterial toxins [10]. Analysis of the chromosome sequence of the *B. anthracis *Ames strain revealed a variety of potential virulence-enhancing factors, including collagenases, phospholipases, hemolysins, proteases and other enterotoxins identified based on their sequence homology with pathogenic factors in other bacterial species [11]. The *B. cereus *group, which includes *B. anthracis, B. thuringiensis and B. cereus*, has an expanded number of predicted secreted proteins relative to nonpathogenic *B. subtilis *[11]. These *B. cereus *group-specific genes represent the ancestral adaptations to a pathogenic lifestyle by the common ancestor, which was quite similar to *B. cereus*. Our attention was attracted to the group of proteases that are encoded on the *B. anthracis *chromosome, shared in common with *B. cereus *but absent or relatively rare in the genomes of nonpathogenic bacteria, such as *B. subtilis *and *B. halodurans*. A large number of these proteases fall into clan MA (classified according to the MEROPS system [12]), which among others includes thermolysin-like enzymes of the M4 family. Metalloproteases (MPs) from several bacterial species belonging to this family are capable of causing massive internal hemorrhages and other life-threatening pathologies [10,13-16].

Whole genome analyses also indicated collagenolytic proteases of the M9B family as potentially having pathogenic functions. Eleven protease families are present in *B. anthracis *and *B. cereus *but absent in *B. subtilis*. Six of the eleven subfamilies encode MPs. Three of the latter, namely M6, M9B, and M20C subfamilies, are encoded on the bacterial chromosomes. Members of the M6 peptidase family are usually annotated as "immune inhibitors" because in *B. thuringiensis *they can inhibit the insect antibacterial response [17]. The M20C peptidase subfamily represents exopeptidases [18] that are the unlikely cause of tissue destruction or internal bleeding. Based on the above analysis, this study focused on the M4 family thermolysin/elastase-like neutral proteases and the M9 family collagenases as the candidate virulence-enhancing factors of *B. anthracis *Aimes strain.

### Hemorrhagic, caseinolytic and gelatinolytic activities of anthrax proteases

**Figure 1:**
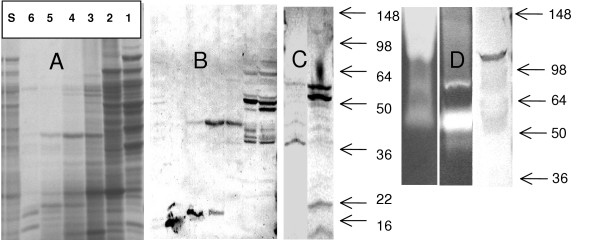
SDS-PAGE of BACS fractions separated on size exclusion column (A) and Western blots: fractions (A) with specific antisera a-M4EL (B), BACS with a-M4AC (C, left lane), BACS with a-M4EP (C, right lane), BACS with a-M9Coll (D, right lane), and zymograms of caseinolytic and gelatinolytic activities of BACS (D, left and center lanes, correspondingly). Molecular masses (KDa) of the marker proteins are indicated by arrows. In A, s denoted BACS, and numbers above correspond to column fractions. In zymogram gels 3 μl of BACS were loaded.

The proteins secreted by three *Bacillus *species (*B. anthracis, B. cereus and B. subtilis*) into culture media were prepared by successive steps of inoculation of the culture media with spores, overnight incubation at 37°C, removal of bacterial cells by centrifugation, sterilization of the supernatant by filtration through 0.22 μ filter and further 50-fold concentration using ultrafiltration devices Amicon Ultra 15 (Millipore, MA) with a 10 KDa cutoff size. The SDS-PAGE gel separation (Fig. 1A) demonstrates the protein content in the concentrated *B. anthracis *culture supernatant (designated as BACS) used in our animal tests. Similar procedures were used to prepare culture supernatants for *B. cereus *ATCC #11778 and *B. subtilis *ATCC #23857 (designated BCCS and BSCS, respectively). Proteolytic activities of BACS are readily detected by zymography using casein or gelatin (denatured collagen) (Fig. 1D). A major band of gelatinase activity corresponds to molecular mass of about 100 KDa, whereas a collagenase activity is represented by about 55 KDa proteins.

**Figure 2:**
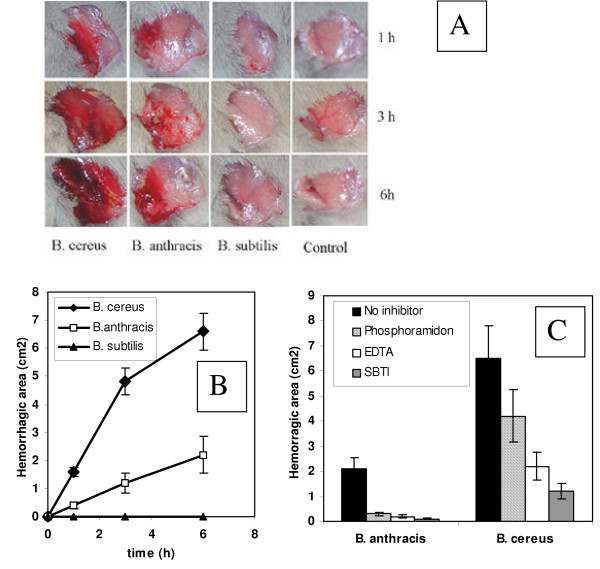
Hemorrhagic activity of culture supernatants (A), its graphic representation (B) and inhibition with chemical inhibitors (C). 100 μl of secreted proteins (20 to 100 μg) were subcutaneously (sc) injected into the femoral artery region for observation of hemorrhagic changes. The fur over the femoral artery region was removed to allow observation of a 1.5 to 2.5 cm^2 ^area of skin. Control mice were administered equal amount of phosphate-buffered saline. Error bars indicate standard deviations.

The concentrated culture supernatants were tested in mice. Upon subcutaneous administration, mice developed hemorrhages of different intensity within several hours (Fig. 2A, B). BCCS showed the highest activity followed by BACS, while BSCS was completely inactive. Chemical inhibitors such as phosphoramidon (potent chelating inhibitor of thermolysin and other M4 bacterial metallo-endopeptidases [19]), EDTA (specific for a broad range of MPs) and soybean trypsin inhibitor (SBTI, reversible competitive inhibitor of trypsin and other trypsin-like proteases such as chymotrypsin, plasmin and plasma kallikrein [10]) effectively abrogated the hemorrhagic affect of BACS (Fig. 2C). The murine serum raised against the recombinant protein corresponding to the mature form of the M4-type thermolysin-like neutral protease of *B. anthracis *(gene identification number, BA 3442 according to [11]) was also effective in suppressing the hemorrhagic effect in the skin test. In negative control experiments, neither naïve murine serum nor three irrelevant murine sera against *B. anthracis *candidate pathogenic factors, hemolysins O, A and B [20] showed anti-hemorrhagic activity (data not shown). Additional control experiments demonstrated that under the conditions of our test the hemorrhagic activity of thermolysin from *B. thermophilicus *was detectable in a dose range from 10 to 100 μg, similar to that for BACS. In contrast to BACS, the inhibitors displayed only partial protection in the case of BCCS (Fig. 2C). Overall, these results correlate with the experimental data that culture supernatants obtained from fully-virulent toxigenic strain of *B. anthracis *were less toxic to mice compared to *B. cereus *ones [9,21].

### Generation of antibodies against *B. anthracis *MPs

Obvious complexity of the BACS protein composition prompted us to develop specific means of detection and inhibition of its components. For this purpose several high-titer immune sera were raised in mice and rabbits using the antigens listed in Table 1. The sera were used in Western blots of BACS proteins. When the proteins were directly separated in the SDS-PAGE for subsequent transfer to the nitrocellulose membrane, the resulting blots were of low intensity indicative of proteolytic degradation during the electrophoresis (Fig. 1A and 1B, left lanes). In order to avoid this complication the BACS was fractionated according to the molecular masses of its components on the Superdex size exclusion column in the presence of EDTA as a chelating agent. Analysis of the column fractions in SDS-PAGE showed a complex pattern of proteins bands (Fig. 1). Multiple proteins with a broad spectrum of molecular masses seem to be highly associated and migrate through the column as high molecular mass complexes. Several factors, such as the presence of multiple precursor and mature protein forms resulting from specific proteolytic maturation, along with nonspecific proteolytic products, can potentially contribute to the complexity of the fractions' composition. Western blot experiments with column fractions revealed several discrete bands recognized by antibodies (Fig. 1). The M4 proteases are represented by several major bands at about 50 KDa, as well as by the bands at about 40 and 20 KDa. These bands probably correspond to different maturation forms of proteases, including the enzymes lacking signal peptides, and mature enzyme forms. The M9 collagenases are detected as a major band with a molecular mass of about 98 kDa which is close to the estimated mass of the pro-enzymes, however the major gelatinase enzymatic activity corresponds to the 55 kDa proteins in the BACS.

### Acute toxicity of *B. anthracis *culture supernatants

**Figure 3:**
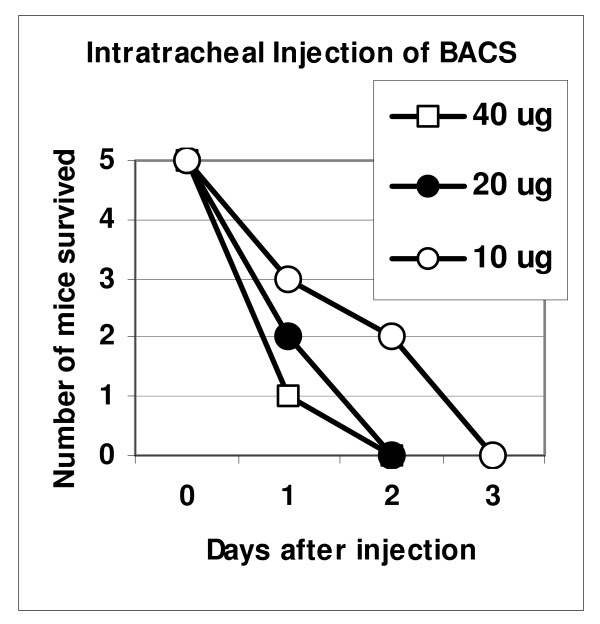
Survival of mice upon intratracheal injection of BACS.

Although bacterial proteases are well known pathogenic factors, little information is available regarding their acute toxicity. We tested BACS in mice using intratracheal administration into the lungs because hemorrhagic mediastinitis and lung edema typically precede the lethal outcome in late anthrax. Therefore, lung damage may be considered as a probable death-causing factor. Mice were given different doses of BACS (10 μg to 40 μg of total protein) and were observed daily for lethality. Fig. 3 shows that all mice challenged with different protein doses died within 2 to 3 days, while the highest dose caused 80% mortality on day 1. For histopathological examination mice were given 100 μg of BACS protein. All animals died within 3 to 4 hours. Postmortem harvested lungs revealed focal intraalveolar acute hemorrhage, which was from minimal to moderately severe with no endothelial cell damage or vasculitis, and mild patchy congestion of medium-size blood vessels. There was evidence of focal platelet accumulation located within areas of hemorrhage or within vessels. In a control experiment, lethal toxin at a comparable dose (100 μg LF, 100 μg PA) caused neither mortality nor hemorrhage, and in fact, produced no significant identifiable histopathological changes. In an additional control experiment, 100 μg of BSCS protein demonstrated no lethality (data not shown).

### Protection of mice against anthrax using protease inhibitors

Effective suppression of the hemorrhagic activity of BACS with chemical inhibitors prompted us to test their protective effect against *B. anthracis *infection. Two common chemical inhibitors were tested in this study: phosphoramidon and 1, 10-phenanthroline. As discussed above, phosphoramidon inhibits the hemorrhagic effect of BACS. It is a potent inhibitor of thermolysin and other bacterial metallo-endopeptidases, but it does not inhibit trypsin, papain, chymotrypsin or pepsin and weakly inhibits collagenase [19]. Phenanthroline is a potent chelating inhibitor of M4 MPs, such as pseudolysin, as well as matrix MPs [10]. We first tested the above inhibitors for their capacity against the bulk of gelatinase and caseinase activity of BACS in vitro. It was found that up to 1 mM phosphoramidon did not inhibit the BACS gelatinase activity, while 50% of caseinolytic activity was inhibited at the concentration of 0.1 mM (IC_50_). In contrast, phenanthroline was more potent with gelatin as substrate (IC_50 _0.01 mM), compared to casein (IC_50 _0.1 mM).

The *B. anthracis *delta Ames strain is avirulent in mice, and therefore for the spore challenge experiments we used a toxigenic Sterne strain. We have previously reported the successful application of an adjunct therapy against anthrax infection targeting both bacterial multiplication and host response to infection by using a combination of antibiotic and caspase inhibitors [22]. The same principle was used in the current study because inhibition of secreted pathogenic factors is not expected to directly interfere with bacterial multiplication and therefore may not be fully protective. In order to target both the bacteria and the proteolytic factors we used a combination therapy where antibiotic administration was complemented by the administration of a protease inhibitor. We were also interested in the efficacy of delayed treatment initiated after certain periods of time following spore challenge. It is a practically relevant scenario because patients generally seek medical help after the onset of symptoms, and in other patients treatment begins after a certain period of time required to confirm the exposure. In addition, a delayed ciprofloxacin therapy in murine model is only partially protective when currently recommended human antibiotic doses (adjusted for body weight) are used in mice [22]. These conditions of treatment allowed us to test if a combination approach could lead to a synergistic enhancement in survival.

**Figure 4:**
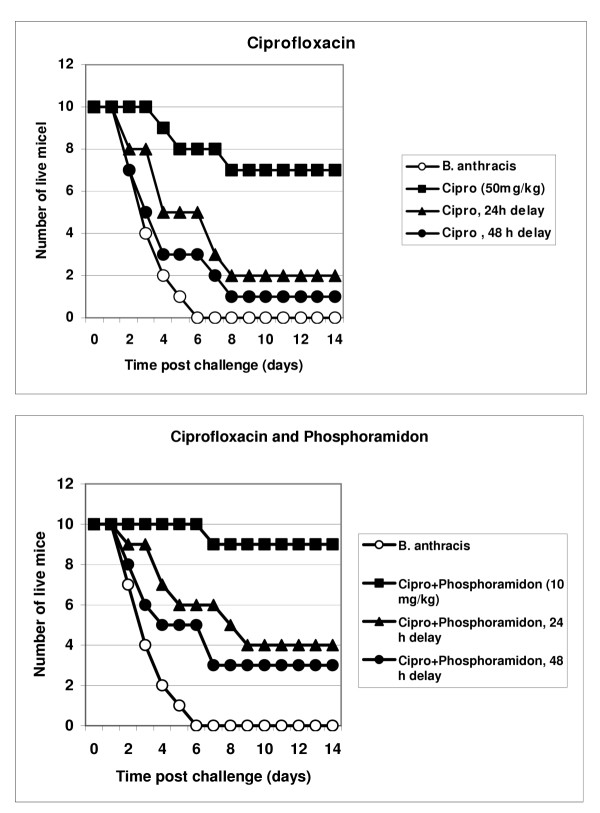
Protection of mice against *B. anthracis *(Sterne) infection by administration of ciprofloxacin and its combination with phosphoramidon for 10 days beginning 24 h and 48 h post spore challenge.

**Figure 5:**
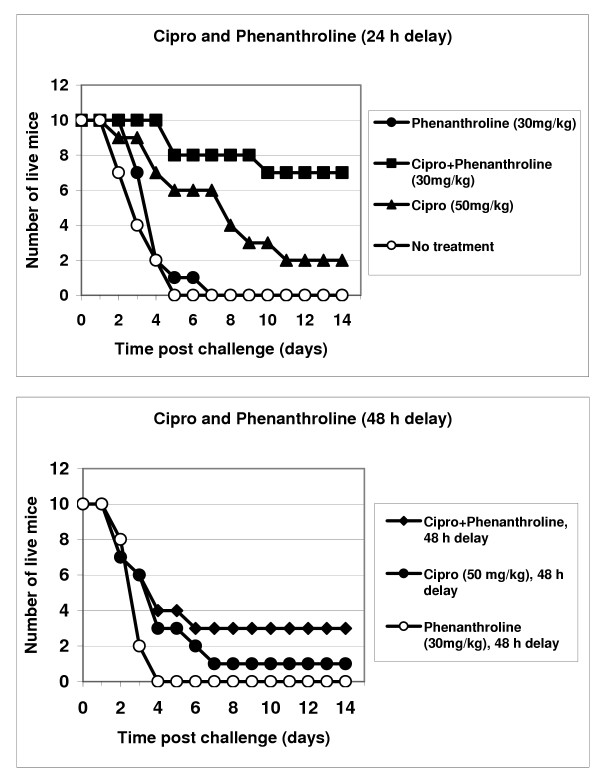
Protection of mice against *B. anthracis *(Sterne) infection by administration of ciprofloxacin or its combination with phenanthroline for 10 days beginning 24 h and 48 h post spore challenge.

Results of three independent experiments are presented in Figs. 4 and 5. Mice were challenged intraperitoneally (i.p.) with about 1 × 10^7 ^of *B. anthracis *Sterne spores. Treatment with a single daily dose of ciprofloxacin (50 mg/kg, i.p.) began immediately after challenge, as well as at 24 h or 48 h post challenge, and continued for 10 days. In our conditions the ciprofloxacin treatment initiated immediately after spore challenge was only 70% effective in preventing death. The survival rate after a 24 h delay in antibiotic administration produced a sharp decline to 20% but remained statistically reliable (compared to untreated group, p = 0.015). After a 48 h delay the antibiotic was completely ineffective (p = 0.23). The inhibitor treatment without antibiotic was not able to improve survival, however the combination of ciprofloxacin with inhibitors displayed a synergistic increase in protection, especially notable in the case of phenanthroline. The group receiving phenanthroline/ciprofloxacin treatment delayed by 24 h, demonstrated 70% protection of animals, compared to only a 20% survival in the group with ciprofloxacin alone (p = 0.03 for these groups). The 48 h-delayed regimen resulted in a statistically reliable 30% protection (relative to the untreated spore-challenged group, p < 0.05), in contrast to ciprofloxacin alone (relative to the untreated spore-challenged group, p = 0.23). There is a similar trend in the efficacy of the combination phosporamidon/ciprifloxacin therapy, compared to ciprofloxacin alone, however the observed differences are less reliable (p > 0.05).

### Protection of mice against *B. anthracis *using anti-protease sera

**Figure 6:**
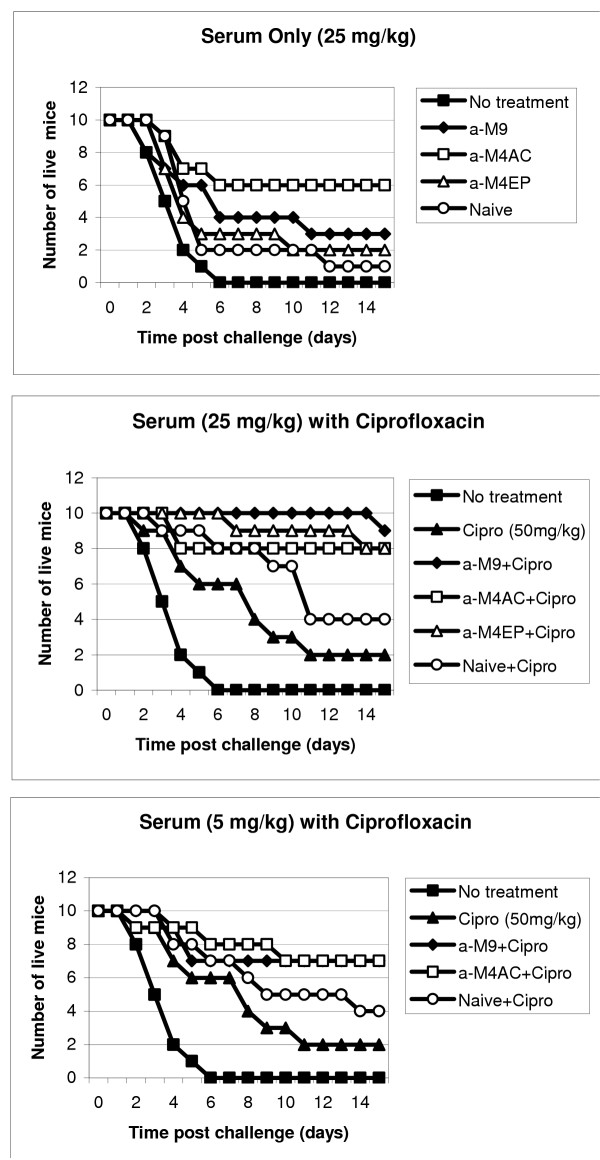
Post exposure efficacy of hyperimmune rabbit sera in mice challenged with *B. anthracis *(Sterne). Treatment with sera and ciprofloxacin was initiated 24 h post exposure and continued for 10 days once daily.

As in the experiments with inhibitors, mice were challenged intraperitoneally (i.p.) with about 30 LD50 of *B. anthracis *Sterne spores. Treatment with a single daily dose of ciprofloxacin (50 mg/kg, i.p.) began at 24 h post challenge and continued for 10 days. The immune sera (each pulled from two rabbits) were administered once daily at a concentration of 25 mg/ml (i.p.). The sera displayed substantial differences in their protective effect (Fig. 6). The anti-M4 serum against the epitope(s) of the active center displayed the highest protection (60%; p = 0.038 compared to naïve serum). The rest of the immune sera, namely the anti-collagenase serum anti-M9Coll and the anti-neutral protease serum anti-M4EP behaved similar to the naïve serum. All three latter sera demonstrated a borderline protection from 10% to 30%, compared to untreated mice (p = 0.028, 0.084, and 0.052, respectively), however the difference in survival between them was statistically unreliable (p > 0.41).

A combination treatment with both antibiotic and all studied immune sera, administered at the dose of 25 mg/kg was strongly synergistic and protected from 80 to 100% mice with high statistical significance (p = 0.0024, 0.0058, and 0.016 for anti-M9, anti-M4EP, and anti-M4AC, correspondingly, compared to the effect of naïve serum-antibiotic combination). A lower serum dose (5 mg/kg) showed a similar pattern of protection, however the effect of combination treatment was reduced to 70%. Some synergistic protection was also noticed in the case of naïve serum (perhaps due to its natural bactericidal properties), although it is statistically unreliable compared to antibiotic-alone group.

## Discussion

In order to be highly virulent, any pathogenic microbe is required to possess the means to effectively establish and further propagate the infectious process. Distinct virulence factors may be necessary to fulfill these requirements at different stages of the disease. *B. anthracis *is a recently emerged highly virulent pathogen, which acquired two plasmids pXO1 and pXO2 compared to the genetically similar but opportunistic pathogen *B. cereus*. These plasmids encode for the lethal toxin (LT) and capsule genes, respectively [4]. LT is a secreted protein, which has long been considered as a major late virulence factor. It appears in the circulation at the septisemic stage of the disease shortly before death [23,24]. LT is the only anthrax protein, which was found toxic in experimental animals [23,24], and this discovery essentially abrogated further research efforts on other potential virulence factors. However, it has long been known that systemic toxicity of LT is low [24,25], and that the histopathology of LT intoxication differs considerably from that found in clinical anthrax infection [5,6]. It is especially notable that postmortem examination of victims from the Sverdlovsk accident and those autopsied following the 2001 U.S. anthrax attacks [2,4] revealed hemorrhagic thoracic lymphadenitis and necrotizing hemorrhagic mediastinitis in all patients. About half of the Sverdlovsk victims additionally had hemorrhagic meningitis [2]. These severe life-critical symptoms had not been noticed in the intoxicated animals.

Recent data, however, suggest a new important role of LT as a disease-establishing virulence factor playing an immunosuppressive role within alveolar macrophages at the early stages of inhalation anthrax [for review, see ref. [26]]. The immunosuppressive capacity of LT is limited, and therefore LT may not completely inhibit the pro-inflammatory host response [27]. Nevertheless, the LT activity does help anthrax spores to survive in the hostile environment, which contributes to the decrease in the infectious dose. It has been demonstrated that LT caused apoptotic death of macrophages and that its inhibition decreased survival of *B. anthracis *spores engulfed by macrophages [28]. These data suggested that the major roles of LT and ET might actually be to provide a more hospitable environment for the pathogen intra-phagocytic survival. This hypothesis helps explain the low virulence of the plasmid-free strains of both *B. anthracis *and *B. cereus *upon spore challenge [29] in spite of the highly toxic proteolytic capacity of these species.

While only fragmented data have been reported on the existence of *B. anthracis *chromosome-encoded virulence factors [20,30,31], it is well established that *B. cereus *produces a variety of pathogenic determinants, including a necrotizing enterotoxin, an emetic toxin, extracellular proteases, phospholipases and hemolysins [32]. *B. cereus *is capable of causing serious and sometimes lethal infections such as sepsis, pneumonia, meningitis, endocarditis, wound and ocular infections, especially in immunocompromised individuals [32-35]. A highly virulent isolate of *B. cereus *has recently been identified which contains a plasmid 99.6% similar to pXO1 [36]. This finding is consistent with the point of view that the *B. cereus *genetic background is sufficient for high virulence when it is complemented with an infection-establishing virulence factor such as LT. Complete sequencing of the *B. anthracis *and *B. cereus *genomes confirmed their close relationship suggested previously [37] and allowed us to suggest new candidate virulence factors for *B. anthracis*, specifically the proteases of the M4 and M9 families. Structurally similar proteolytic factors in other pathogenic microorganisms are known to be involved in inactivation of complement factors [38], cleavage of serum protease inhibitors [39], activation of blood coagulation system [40], invasiveness into the host tissue [41], and development of hemorrhages [13].

We demonstrate here that secreted metalloproteases (MPs) of *B. anthracis *can digest protein substrates such as casein and gelatin *in vitro*, and can induce a hemorrhagic process in our test subjects, *in vivo*. Both of these activities are inhibited by chemical inhibitors of M4 and M9 MPs, such as EDTA, phosphoramidon and 1, 10-phenanthroline. Consistent with this, the hyper-immune mouse serum against M4 family thermolysin/elastase-like enzyme is capable of inhibiting the hemorrhagic effect of BACS (we currently investigate the *in vitro *inhibiting activity of the hyper-immune sera used in this report). The tissue-damaging action of this type of enzymes is well known [10]. For example, pseudolysin, an elastase of *Pseudomonas aeruginos*a, destroys arterial elastic laminae in systemic infection, causes lung damage with hemorrhages and necrosis, and induces septic shock through activation of the host kinin cascade [42-44].

In the present study we used an intratracheal (i.t.) administration to demonstrate that tissue destructive and hemorrhagic properties of BACS could cause a lethal effect at 0.5 to 3 mg/kg doses of total protein (10 to 60 μg per 20 g DBA/2 mouse) within a few days or even hours (Fig. 3). Histopathological examination confirmed our observations of life-threatening severe bleeding upon administration of BACS. It has been reported that *P. aeruginosa *elastase induced an immediate lethal shock in guinea pigs upon an i.v. injection at a similar dose of 1.2 mg/kg [44]. Compared to BACS, the LT is non-toxic upon an i.t. administration. There is no mortality in a control group of LT-treated mice (200 μg/mouse, i.t.). Recently, the toxicity of highly purified LT was re-evaluated in BALB/CJ and C57BL/6J mice, and it was found that doses from 5 to 12.5 mg/kg (i.v.) were required for up to 90% mortality in 5 days [6]. We conclude that secreted proteins of *B. anthracis*, in addition to LT and ET, have high pathogenic potential and should be considered as important virulence factors.

We have previously suggested a combination antibiotic-antitoxin approach to anthrax therapy and for the first time demonstrated an increased efficacy of ciprofloxacin treatment in a murine model when it was combined with a caspase inhibitor administration [22]. Up to now, there has been no other report on any anti-LT treatment during the anthrax infectious process. In the present study, we used two new combination therapies, namely the antibiotic-protease inhibitor and the antibiotic-antiserum ones. Both of them also proved beneficial, compared to antibiotic alone. It is especially important to note that we used the delayed treatment regiments, which are inherently less effective, compared to prophylactic drug administration.

Currently, a considerable effort is directed towards development of specific LT inhibitors (see for example [45-47]) while their efficacy in the treatment of the infectious process has not been reported. In contrast, the inhibitors we used (phosphoramidon and phenanthroline) to model anthrax therapy are not considered as LT inhibitors [47], however in our experiments both of these substances increased survival during ciprofloxacin therapy, which was initiated at 24 h and 48 h post challenge. The phenanthroline-ciprofloxacin combination administered 24 h post challenge protected 70% of the mice, compared to 20% for antibiotic alone. Late stages of anthrax are especially difficult to treat [4]. In our model the 48 h post challenge time approximately correlates with the period of typical overt anthrax symptoms in patients because 30% of mice die within the next 24 h, a situation that is similar to that clinically observed in natural human infection. In these circumstances, our therapy with phenanthroline-ciprofloxacin was 30% protective, while ciprofloxacin alone was ineffective. It is worth pointing out that the high extent of protection (70% after 24 h delay) conferred by a combination of antibiotic with phenanthroline argues in favor of the substantial role of secreted proteases as death-causing factors, although their contribution to mortality relative to LT is currently unknown. We have previously reported that in similar experimental conditions the caspase inhibitor YVAD, capable of protecting macrophages against LT-induced apoptosis, improved survival of DBA/2 mice by 30%. One may therefore expect that a triple component therapy, such as ciprofloxacin-phosphoramidon-caspase inhibitor, might be completely protective. Experiments in this direction are in progress. In connection with the question on the role of LT, we also currently investigate a spectrum of neutralizing activity of immune sera used in this study.

## Conclusion

Overall, our data demonstrate that *B. anthracis *cultivated in culture media secrets a number of proteolytic virulence factors, including those with hemorrhagic, caseinolytic and gelatinolytic activities. These factors in most respects are distinct from LT, including the mode of their expression under aerobic conditions (LT requires bicarbonate for its expression in vitro [48]), their molecular targets, as well as a high virulent potency upon intratracheal administration. Chemical inhibitors of these factors as well as immune sera raised against them in rabbits demonstrate a substantial protective efficacy in combination with antibiotic therapy. Our findings outline a new direction in the development of anthrax therapeutic approaches, and close a substantial gap between the understanding of anthrax molecular pathology and the most prominent clinical features of its infectious process. Complexity of the BACS composition with regard to the number and specificity of proteolytic enzymes suggests a multitude of their potential virulent mechanisms that need to be explored further.

## Competing interests

SP, TP, SH, KA, KJF, CB and VC are co-authors of pending patent applications related to the content of the manuscript. The authors have also received salaries from George Mason University and/or Advanced Biosystems, Inc., both of which hold an interest in these patents. The authors declare they have no other competing interests.

## Authors' contributions

SP developed the research plan, directed experimental work and was principal writer of the manuscript. TP analyzed the protease sequences, designed peptides for immunization, and carried out the *in vitro* studies. SH carried out all animal experiments and participated in the in vitro characterization of culture supernatants. RW and RM conducted a histopathological evaluation of tissue specimens. KF carried out bioinformatics analyses and participated in manuscript preparation. KA conceived of the study, developed its initial design, along with CB and VC participated in research coordination and helped to draft the manuscript. All authors read and approved the final manuscript.

## Pre-publication history

The pre-publication history for this paper can be accessed here:


